# The Pros and Cons of Minimally Invasive Surgery Versus Open Surgery for Inguinal Hernia Repair: A Narrative Literature Review

**DOI:** 10.7759/cureus.95217

**Published:** 2025-10-23

**Authors:** Sourav S Panikkassery, Jewel M Kenneth, Raghavendra Bhat

**Affiliations:** 1 General Surgery, Sheikh Khalifa Medical City, Abu Dhabi, ARE; 2 General Surgery, Saqr Hospital, Ras Al Khaimah, ARE; 3 Internal Medicine, Ras Al Khaimah Medical and Health Sciences University, Ras Al Khaimah, ARE

**Keywords:** inguinal hernia repair, inguinal hernia surgery, laparoscopic hernia repair, laparoscopic tapp repair, minimally access surgery, open inguinal hernia repair

## Abstract

The choice between minimally invasive laparoscopic and open surgical approaches for inguinal hernia repair significantly impacts postoperative outcomes, particularly in terms of pain management, recovery time, and patient satisfaction. This literature review synthesizes findings from multiple studies to provide a comparative analysis of these two techniques. Evidence consistently demonstrates that laparoscopic repair demonstrates clear advantages, with patients using 42% fewer analgesics in the first 48 hours, returning to light activity in 8 days compared to 14 days for open repair, and to full activity in 13.6 versus 19.8 days. Furthermore, hospital stays are shorter (1-2 days vs. 2-4 days), and overall satisfaction is higher (88.7% vs. 79.3%). Additionally, complication profiles also differ; laparoscopic repair is linked to lower rates of wound infection, hematoma, and chronic pain, though seroma formation is somewhat higher (15.8-22%). However, laparoscopic techniques are associated with higher upfront costs and require greater surgical expertise, leading to longer operative times, particularly for novice surgeons. While open surgery remains a viable option for certain patient populations, such as those unable to undergo general anesthesia or with complex hernias, laparoscopic repair is increasingly regarded as the superior approach for most cases. This review highlights the clinical and economic implications of these techniques, advocating for patient-centered decision-making to optimize outcomes. With advancements in surgical training and cost management, the benefits of laparoscopic repair can be extended to a broader patient population.

## Introduction and background

Inguinal hernia repair is one of the most frequently performed surgical procedures worldwide, with approximately 20 million repairs conducted annually [1]. Over the years, surgical techniques have evolved, offering patients multiple options, including conventional open repair techniques such as Lichtenstein and minimally invasive laparoscopic approaches such as totally extraperitoneal (TEP) and transabdominal preperitoneal (TAPP) repair. The debate over the optimal surgical technique centers on key factors such as postoperative pain, recovery time, and long-term outcomes. While both methods aim to effectively repair the hernia and prevent recurrence, they differ significantly in their impact on the patient’s recovery experience and overall quality of life [2,3].

Postoperative pain is a critical determinant of recovery success, influencing a patient’s return to daily activities and overall satisfaction with the surgical experience [4]. Additionally, the length of recovery time can have substantial socioeconomic implications, particularly for working-age patients. Studies have shown that minimally invasive techniques often lead to quicker recovery and fewer complications, but these benefits may be offset by higher costs and the need for advanced surgical expertise [1].

This literature review aims to provide a comprehensive comparison of laparoscopic and open surgical techniques for inguinal hernia repair, focusing on postoperative pain, recovery time, complications, and patient satisfaction. By synthesizing data from recent studies, this review seeks to inform clinical decision-making and highlight the advantages and limitations of each approach.

## Review

Methodology

This narrative review was conducted following the Scale for the Assessment of Narrative Review Articles (SANRA) guidelines. The methodology involved systematically gathering, critically evaluating, integrating, and presenting the existing literature to provide a comprehensive overview of current evidence.

Electronic databases, including PubMed, MEDLINE, and Embase, were searched for studies published up to March 2025. Search strategies combined keywords related to inguinal hernia, various repair options, and the various parameters affecting the patients, which included “inguinal hernia,” “inguinal hernia repair,” “laparoscopic surgery,” “open surgery,” “minimally invasive techniques,” “postoperative pain,” “recovery time,” “complications,” “long term outcomes,” “patient satisfaction,” and “quality of life,” using Boolean operators AND and OR to optimize search precision and inclusivity. Reference lists of key publications were also reviewed to ensure comprehensive coverage.

While robotic-assisted repair appeared in our search results, the primary focus of this review was on laparoscopic surgery versus open surgery. Therefore, robotic techniques are discussed briefly as an emerging modality, rather than being systematically analyzed alongside the main comparators.

Inclusion and Exclusion Criteria

To ensure the relevance and quality of evidence, studies in this literature review were included based on specific eligibility criteria. Eligible studies focused on adult patients aged 18 years or older who underwent inguinal hernia repair. The primary intervention of interest was laparoscopic or other minimally invasive surgical techniques, which were compared directly to conventional open surgical repair. Studies were required to report at least one relevant clinical outcome, such as postoperative pain, recovery time, complication rates, recurrence, patient satisfaction, or quality of life. A range of study designs was considered, including randomized controlled trials (RCTs), prospective or retrospective cohort studies, systematic reviews, and meta-analyses. Only articles published in peer-reviewed journals and available in English were included.

Studies were excluded if they lacked original data, such as case reports, conference abstracts, letters to the editor, or narrative reviews. Research that did not include a direct comparison between laparoscopic and open techniques was also excluded, as were studies focused on pediatric populations, animal models, or laboratory-based experiments. Articles without full-text availability were not considered.

Each study was critically appraised for methodological rigor, design, and sample size. Thematic analysis was used to organize findings into key domains, including pain outcomes, recovery time, postoperative complications, patient satisfaction, and economic implications.

Anatomy and herniation

**Figure 1:**
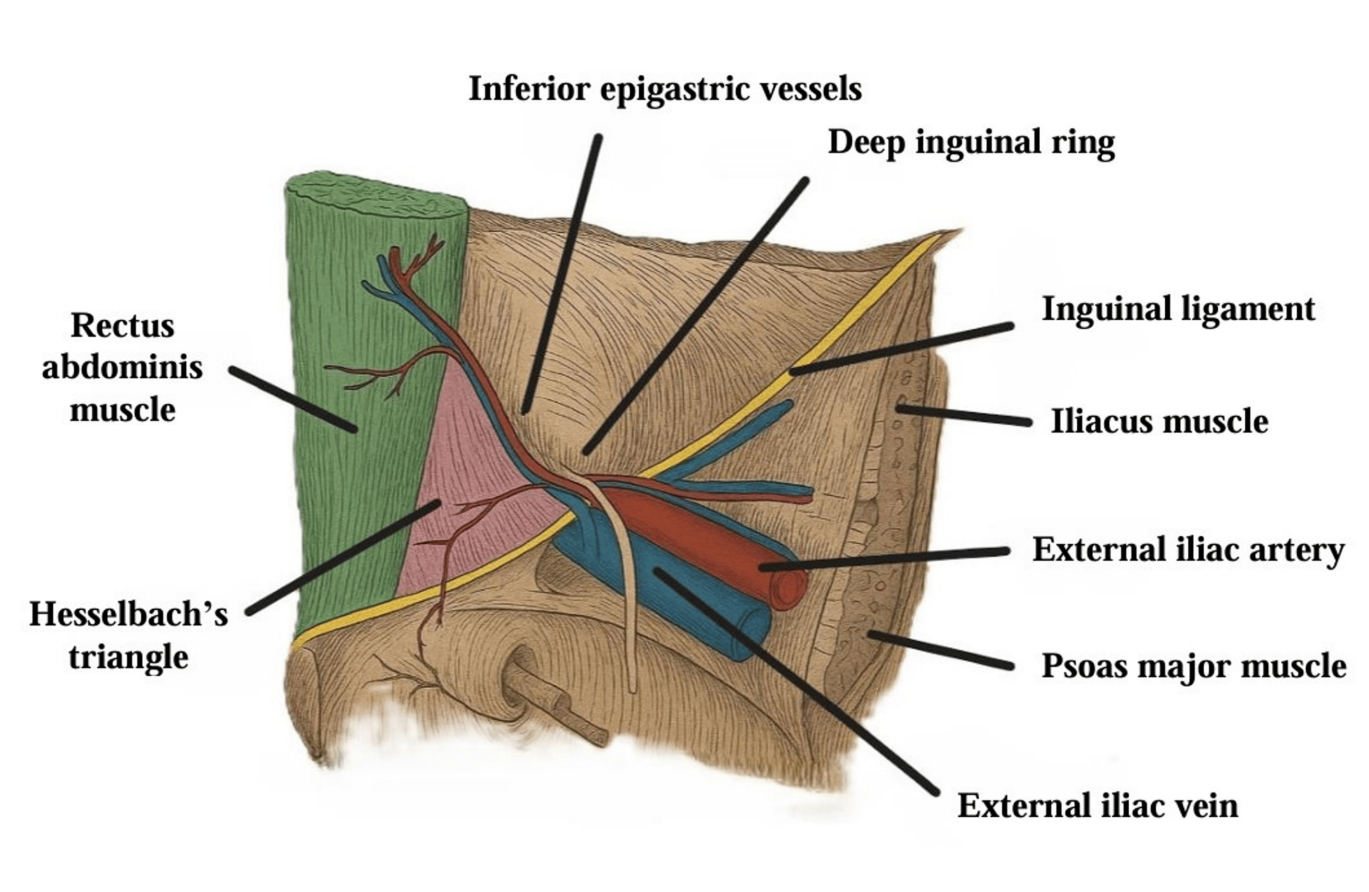
Anatomy of the inguinal region. Authors’ own image.

The inguinal canal, located in the lower anterior abdominal wall, extends from the internal to the superficial inguinal ring and contains the spermatic cord in males and the round ligament in females. The integrity of the abdominal wall relies on the proper alignment of the inguinal canal, the transversalis fascia, and the sphincter-like function of the internal ring [1]. Understanding the canal’s anatomy is crucial for hernia formation: lateral hernias emerge from the internal inguinal ring, often through a patent processus vaginalis, while medial hernias occur when the transversalis fascia in Hesselbach’s triangle weakens, allowing abdominal contents to herniate [1,2] (Figure 1).

Etiology

Inguinal hernias result from both congenital and acquired factors, with most adult cases considered acquired. Genetics plays a role, as individuals with a family history are at a higher risk. Conditions such as chronic obstructive pulmonary disease, Ehlers-Danlos syndrome, and Marfan syndrome further increase susceptibility [2]. Increased intra-abdominal pressure due to obesity, chronic cough, heavy lifting, or straining from constipation also contributes. While the role of smoking in primary hernia formation is unclear, it increases the risk of recurrence after repair [3].

Epidemiology

Inguinal hernia repair is common, with roughly 800,000 surgeries annually in the United States, representing 75% of abdominal wall hernias [2]. The incidence has a bimodal age distribution, peaking around ages 5 and 70. Males account for ~90% of cases, while indirect hernias are most frequent, typically on the right side due to delayed closure of the processus vaginalis. Risk factors include advancing age, male gender, and systemic connective tissue disorders. Femoral hernias are less common (3% of cases) and more prevalent in females [2,3].

Pathophysiology

Connective tissue alterations, particularly an imbalance in collagen subtypes, underlie hernia formation. Patients with inguinal hernias have higher proportions of type III collagen, which has lower tensile strength than type I collagen; this imbalance is more pronounced in medial hernias [1]. A patent processus vaginalis is a key risk factor in children, allowing abdominal contents to pass through the inguinal canal. Incomplete obliteration may result from defects in smooth muscle apoptosis or other molecular mechanisms. Research has also explored the role of smooth muscle cells and calcitonin gene-related peptide in regulating processus vaginalis closure [1,3].

Open surgery techniques

Open surgery remains one of the most commonly used methods for inguinal hernia repair. The procedure typically starts with a 5-6 cm incision parallel to the inguinal ligament, through which the surgeon dissects to identify the external oblique fascia [4]. The surgeon then opens this fascia parallel to its fibers to expose the spermatic cord and the hernia sac.

The hernia can be classified as either direct or indirect based on its anatomical location, with direct hernias occurring medially to the inferior epigastric vessels, while indirect hernias pass laterally [4]. The most commonly performed procedure in open surgery is the Lichtenstein tension-free hernioplasty, in which mesh is used to cover the defect in the abdominal wall, reinforcing the inguinal floor and reducing the risk of recurrence [5].

Non-mesh repairs, such as the Shouldice and Bassini repairs, rely on suturing the muscles and fascia to reinforce the inguinal canal [4]. While these methods are effective, they require careful handling of nerves, such as the ilioinguinal nerve, which remains a subject of debate regarding preservation or sacrifice [4]. Open surgery is associated with higher postoperative pain levels and a longer recovery time compared to minimally invasive approaches. Patients are usually instructed to avoid heavy lifting or strenuous activity for at least four to six weeks to prevent recurrence [5].

Laparoscopic surgery techniques

Laparoscopic hernia repair is a minimally invasive technique that uses small incisions and specialized instruments to perform the surgery. This method offers several advantages over open surgery, including reduced postoperative pain, quicker recovery times, and fewer wound complications [6].

Laparoscopic repair involves the insertion of a camera and tools through tiny abdominal incisions, allowing the surgeon to repair the hernia using a mesh, similar to open surgery but with less tissue disruption [6]. This technique requires general anesthesia, specialized training, and access to advanced equipment, making it more expensive and less accessible in some regions [6]. However, laparoscopic surgery is particularly beneficial for patients with bilateral or recurrent hernias, as it provides a more comprehensive view of the entire inguinal canal, allowing the surgeon to address multiple defects in one procedure [6]. The minimally invasive nature of the surgery reduces the length of hospital stays and allows patients to return to their normal activities more quickly than with open surgery [5].

Despite its advantages, the learning curve associated with laparoscopic techniques is steep, and the procedure requires specialized skills that can limit its widespread use in some settings [3].

Robotic surgery techniques

Robotic surgery represents the most advanced form of minimally invasive inguinal hernia repair. This technique utilizes robotic arms controlled by the surgeon, providing enhanced precision, better visualization, and improved dexterity [5]. Robotic surgery allows for complex repairs with greater accuracy, making it particularly useful in cases of recurrent or difficult hernias where precision is critical [6]. The robot’s three-dimensional camera system offers high-definition views of the surgical site, enhancing the surgeon’s ability to navigate delicate structures and perform intricate dissection [6].

Similar to laparoscopic surgery, robotic techniques reduce postoperative pain and shorten recovery times, but they are associated with significant costs and require specialized training and equipment [5]. While robotic surgery offers advantages in terms of precision and outcomes, it is less commonly available due to its higher costs and the need for advanced infrastructure. In settings where robotic equipment is available, it offers an excellent option for patients with complex hernias who might benefit from enhanced accuracy and fewer complications [6,7].

Robotic repair was not included in the main comparative analysis due to the scarcity of robust head-to-head data. Future studies directly comparing robotic, laparoscopic, and open approaches will be valuable to better define their role.

Postoperative pain

Postoperative pain is a primary outcome influencing both patient satisfaction and recovery trajectories following inguinal hernia repair. Evidence across multiple RCTs and meta-analyses consistently shows that laparoscopic repair results in lower pain intensity compared to open repair, particularly in the immediate and short-term postoperative phases. Tanphiphat et al. [8] observed significantly lower pain scores within 24 hours, while Pokorny et al. [9] reported a 42% reduction in analgesic consumption in the first 48 hours. Kozol et al. [10] confirmed these findings using the McGill Visual Analog Pain Scale, showing both lower scores and fewer analgesics required postoperatively.

Patterson et al. conducted a meta-analysis of 21 studies, which demonstrated that mean VAS scores during the first two weeks were significantly lower following laparoscopic repair (2.27, range = 0-4.2) compared with open repair (2.98, range = 0.6-5.2) [7]. Their analysis confirmed this benefit with a mean difference (MD) of -0.69 (95% confidence interval (CI) = -0.78 to -0.61). Between two weeks and six months postoperatively, mean VAS scores remained lower after laparoscopic repair (1.5 vs. 2.3; MD = -0.88, 95% CI = -1.39 to -0.36) [7].

Chronic pain findings are heterogeneous to an extent. Patterson et al. [7] and Shah et al. [11] reported significantly lower chronic groin pain after laparoscopic repair, while Beldi et al. [12] found lower numbness but similar rates of chronic pain compared with open repair. When examined in aggregate, however, the trend favors laparoscopy: at 6-12 months, 20 studies demonstrated a crude incidence of chronic pain of 10.3% for laparoscopy versus 13.4% for open repair (risk ratio (RR) = 0.74, 95% CI = 0.59 to 0.93) [7]. After more than one year, the advantage persisted, with laparoscopic repair associated with lower long-term pain rates (6.6% vs. 9.4%; RR = 0.62, 95% CI = 0.47 to 0.82) [7,8].

Overall, evidence from most studies indicates that laparoscopic repair is associated with reduced acute and late chronic pain, as well as lower rates of numbness and sensory disturbances, although surgeon expertise and fixation method influence outcomes.

Recovery time

The recovery period following surgery is critical for patients’ return to daily life and work. Laparoscopic surgery consistently demonstrates faster recovery times compared to open repair. A systematic review found that laparoscopic procedures allowed patients to resume light activities in a median of 8 days, compared to 14 days for open surgery (p = 0.013) [8]. Similarly, patients returned to full activity sooner after laparoscopic repair, averaging 13.6 days compared to 19.8 days for open repair (p < 0.001) [10].

Hospital stay durations were also shorter for laparoscopic surgery. One study reported that laparoscopic patients were typically discharged after one to two days, whereas open surgery required longer stays of two to four days [9]. Beldi et al. [12] noted reduced numbness in laparoscopy, though some studies suggest chronic pain rates may converge depending on surgeon technique and patient factors. Another analysis highlighted that laparoscopic patients returned to work on average 3.4 days earlier than those undergoing open repair, underscoring the socioeconomic benefits of minimally invasive techniques [13].

Factors contributing to quicker recovery in laparoscopic surgery include reduced tissue trauma, smaller incisions, and lower rates of wound complications. A study comparing laparoscopic TEP and open Lichtenstein repairs revealed that chronic groin pain and prolonged disability were significantly more common in the open group [11].

Complications and long-term outcomes

Minimally invasive approaches generally demonstrate a favorable complication profile, though they are not without challenges. Laparoscopic repairs show lower rates of wound infection, hematoma, and chronic pain compared to open methods [14-16], likely due to reduced tissue trauma and smaller incisions.

Seroma formation is more frequent following laparoscopic repair, with reported rates between 15.8% and 22%, though most cases resolve without intervention [10,11]. Operative times are typically longer during the early learning curve; mean durations for bilateral repairs are around 78 minutes for laparoscopy versus 102 minutes for open repair, while unilateral repairs average 63 and 70 minutes, respectively [13].

Recurrence rates, a key indicator of long-term success, were similar across laparoscopic and open approaches when performed by experienced surgeons. Evidence suggests that recurrence depends more on surgical expertise and mesh fixation quality than on the chosen technique [14].

Overall, laparoscopic repair offers favorable long-term outcomes, with fewer chronic pain-related complications and faster recovery, reinforcing its value as a reliable minimally invasive option.

Patient satisfaction and quality of life

Patient satisfaction is an important determinant of surgical success and is generally higher with laparoscopic approaches. In one study, 88.7% of laparoscopic patients reported being highly satisfied with their procedure, compared to 79.3% of open surgery patients [7]. This satisfaction stems from reduced pain, faster recovery, and improved aesthetics due to smaller incisions.

Quality of life metrics further validate these findings. A prospective study using the Medical Outcomes Study Short-Form 36 questionnaire showed that laparoscopic patients reported better physical functioning and lower pain levels at one month postoperatively compared to open repair patients [15]. Despite these advantages, higher costs and the need for general anesthesia may limit the accessibility of laparoscopic techniques [13].

Economic and societal implications

While laparoscopic repair incurs higher initial costs due to longer operative times and specialized equipment, its societal benefits, such as quicker return to work and fewer long-term healthcare needs, make it cost-effective in the long run [13]. Furthermore, the use of reusable instruments and increased surgeon proficiency can help mitigate the cost differences over time [14].

Table 1 presents a summary of pertinent findings of the included studies. 

**Table 1:** Summary of the findings of the included studies.

Study	Findings
Patterson et al. [7]	Laparoscopic surgery offers less postoperative pain, quicker recovery, and higher satisfaction compared to open surgery
Beldi et al. [12]	Laparoscopic repair results in lower numbness but similar chronic pain incidence compared to open repair
Tanphiphat et al. [8]	Laparoscopic repair provides less immediate postoperative pain but is costlier and requires longer operative time
Kozol et al. [9]	Laparoscopic repair shows significantly less postoperative pain and analgesic use
Pokorny et al. [15]	Transabdominal preperitoneal (TAPP) laparoscopic technique results in lower pain levels and similar quality of life (QoL) to open techniques
Lawrence et al. [17]	Laparoscopic repair offers better early postoperative pain outcomes but higher complications
Wellwood et al. [18]	Laparoscopic repair reduces recovery time and pain but is more expensive
Shah et al. [10]	Total extraperitoneal (TEP) repair leads to reduced pain, quicker recovery, and fewer complications than Lichtenstein
Castro et al. [19]	Laparoscopic techniques show quicker recovery and lower morbidity, with similar long-term QoL outcomes
Knight et al. [20]	Both minimally invasive surgery and open repairs show similar postoperative opioid requirements, supporting conservative prescribing
Gritsiuta et al. [21]	Robotic-assisted laparoscopic repair reduces pain, recovery time, and complications compared to open surgery
Cavazzola et al. [14]	Laparoscopic repair results in less chronic pain, quicker recovery, but requires greater surgical expertise
Zhu et al. [22]	Laparoscopic surgery offers lower pain and faster recovery, with patient-specific factors influencing approach
Tolver et al. [23]	Non-restrictive recovery guidelines shorten recovery without compromising safety
Aly et al. [16]	TEP provides quicker recovery and less pain, but Lichtenstein repair is cost-effective for higher-risk patients
Moreno-Suero et al. [11]	Laparoscopic methods reduce hospital stay, infection rates, and complications compared to open surgery
Winslow et al. [13]	TEP repair offers reduced pain, numbness, and faster recovery but more fluid collections than open repair

Limitations

The key limitations of the included studies are the heterogeneity of study designs, variability in outcome reporting, small sample sizes, and short follow-up periods. Additional limitations include potential publication bias, inconsistent reporting of confounding factors such as patient comorbidities or variations in surgical technique, and the underrepresentation of emerging approaches, such as robotic-assisted repair, which were not systematically analyzed.

## Conclusions

The comparison between laparoscopic and open surgical approaches for inguinal hernia repair highlights the significant advantages of minimally invasive techniques in reducing pain, accelerating recovery, and enhancing patient satisfaction. Multiple RCTs demonstrate reduced short-term pain and analgesic use with laparoscopy, while meta-analyses confirm significantly lower mean VAS scores in the early postoperative phase. Although some individual studies suggest no difference in chronic pain, pooled analyses indicate that laparoscopic repair is associated with reduced rates of both early (6-12 months) and late (>1 year) chronic pain. Furthermore, numbness and paraesthesia are less frequent with laparoscopic repair. While open surgery retains relevance in selected patients, the accumulated evidence supports laparoscopic repair as the superior approach for minimizing postoperative pain and sensory disturbances, thereby improving long-term patient outcomes. Although laparoscopic techniques are associated with higher initial costs and a steeper learning curve, several studies suggest that they may offer potential long-term cost benefits due to shorter hospital stays, faster recovery, and fewer postoperative complications. Patient satisfaction has also been reported to be higher in some studies, with improved physical functioning and quality of life contributing to the perceived advantages of this approach. Nevertheless, the choice of surgical technique should remain individualized, considering patient-specific factors, hernia characteristics, and surgeon experience. Robotic-assisted inguinal hernia repair was identified in a small number of studies, but the evidence remains limited and heterogeneous. For this reason, we summarize it separately as an emerging technique, while the main evidence synthesis focuses on laparoscopic versus open repair. While laparoscopic repair is increasingly recognized as the superior option for inguinal hernia repair, open surgery retains its relevance for certain patient groups and clinical scenarios. With continued advancements in surgical training and resource management, the accessibility and efficacy of laparoscopic techniques will likely expand, cementing its role as the preferred method for hernia repair in most cases.
